# Changes in elbow joint contact area in symptomatic valgus instability of the elbow in baseball players

**DOI:** 10.1038/s41598-021-99193-0

**Published:** 2021-10-05

**Authors:** Kyosuke Numaguchi, Daisuke Momma, Yuki Matsui, Jun Oohinata, Takayoshi Yamaguchi, Nozomu Inoue, Eiji Kondo, Norimasa Iwasaki

**Affiliations:** 1 Faculty of Medicine and Graduate School of Medicine, Department of Orthopaedic Surgery, Hokkaido University, Sapporo, Japan; 2 Center for Sports Medicine, Hokkaido University Hospital, Kita 14, Nishi 5, Sapporo, Hokkaido 060-8638 Japan; 3 Tonan Hospital, Sapporo, Japan; 4 Hanaoka Seishu Memorial Hospital, Sapporo, Japan; 5 Department of Orthopedic Surgery, Rush University Medical Center, Chicago, USA

**Keywords:** Bone quality and biomechanics, Musculoskeletal system

## Abstract

The aim of this study was to evaluate the joint contact area of the dominant side and that of the non-dominant side without valgus instability in symptomatic pitchers. Ten symptomatic elbow medial ulnar collateral ligament (UCL) deficient baseball pitchers participated in this study. Computed tomography (CT) data from the dominant and non-dominant elbows were obtained with and without elbow valgus stress. The CT imaging data of each elbow joint were reconstructed using a 3D reconstruction software package, and the radiocapitellar and ulnohumeral joint contact areas were calculated. The center of the contact area and the translation from the position without stress to the position with valgus stress were also calculated. With elbow valgus stress, the contact area changed, and the center of the radiocapitellar joint contact area translated significantly more laterally in the dominant elbow than in the non-dominant elbow (p = 0.0361). In addition, the center of the ulnohumeral joint contact area translated significantly more posteriorly in the dominant elbow than in the non-dominant elbow (p = 0.0413). These changes in contact areas could be the reason for cartilage injury at the posterior trochlea in pitchers with UCL deficiency.

## Introduction

Enormous valgus forces are generated across the elbow during the acceleration phase of the overhead throwing, which results in tremendous tensile forces on the medial side of the elbow^1^. Particularly, repetitive valgus stress to the elbow joint during throwing motions leads to medial elbow injury. Previous studies reported the term medial elbow stress syndrome^1,2^. Medial elbow stress syndrome includes not only ulnar collateral ligament (UCL) deficiency of the elbow joint, but it also includes the existence of medial olecranon fossa hypertrophy, recognized as valgus extension overload syndrome^3^. Thus, throwing activity changes the joint contact area in the elbow. However, because of the difficulties in measuring the joint contact area directly, the actual joint contact area associated with valgus instability has not been confirmed.

Ahmad et al. characterized the effects of UCL injury on olecranon contact within the trochlea in a cadaver model^4^. Previous studies reported a series of competitive baseball players who underwent isolated resection of symptomatic posteromedial osteophytes and found that 25% developed valgus instability and eventually required UCL reconstruction^5^, and more than 50% of their patients undergoing UCL repair or reconstruction had posteromedial osteophytes^6^. Several cadaver and kinematic studies have analyzed the function of the UCL; however, it is difficult to simulate the actual loading conditions of pitching activities on cadaveric joints. In addition, it is difficult to evaluate the three dimensional (3D) articular contact area in vivo.

Elbow joint contact patterns have been considered to reflect pathological conditions such as UCL tears^7^. Based on this theory, Bey et al. reported that joint contact patterns are not only a more sensitive measurement than conventional kinematics for detecting subtle differences in joint function but they may also provide a more clinically relevant indication of the extent to which a conservative approach or a surgical procedure has adequately restored normal joint function^8^. Therefore, the kinematics of the elbow joint in baseball pitchers with symptomatic elbow valgus instability can be determined by measurement of the elbow joint contact patterns. We hypothesized that the joint contact areas of elbows are changed more with elbow valgus stress in pitchers with symptomatic valgus instability than without instability. The aims of this study were (1) to evaluate the contact area across the elbow joint in symptomatic pitchers with UCL deficiency and (2) to then clarify the changes in the contact area with and without elbow valgus stress.

## Results

### Participants’ demographic characteristics

3D CT image data from elbows of both sides of 10 male pitchers (mean age 25.9 ± 3.9 years) were collected for further analysis. The obtained data showed that there were no apparent differences in mean extension/flexion and pronation/supination between the dominant and non-dominant sides (Table 1).

**Table 1:** Participants’ characteristics.

Case	Age, year	Dominance	Height, cm	Weight, kg	Experience, year	MRI classification	ROM, deg, Dominant (D)/Non-dominant (ND)			
							Pronation	Supination	Extension	Flexion
1	32	R	184	85	25	Distal Complete	62/63	81/84	− 5/1	129/134
2	27	R	176	84	17	Distal Complete	67/68	86/88	0/− 1	134/138
3	25	R	183	85	18	Proximal Complete	68/72	89/91	1/4	140/139
4	23	R	177	71	12	Distal Complete	70/71	91/90	4/7	145/143
5	20	R	169	59	8	Distal Complete	72/73	87/91	6/6	143/145
6	24	R	176	75	15	Proximal Complete	63/69	88/90	− 1/3	137/141
7	23	L	177	87	12	Distal Complete	73/67	90/89	6/7	139/137
8	24	R	177	72	15	Proximal Complete	68/70	91/88	1/1	141/143
9	33	R	181	72	26	Distal Complete	69/72	89/89	4/3	140/141
10	28	R	179	80	20	Distal Complete	71/71	90/89	5/5	140/138
Mean	25.9		177.9	77.0	16.8		68.3/69.6	88.2/88.9	2.1/3.6	138.8/139.9
P value D vs ND							0.3906	0.5495	0.3020	0.5453

### Joint contact area

**Figure 1:**
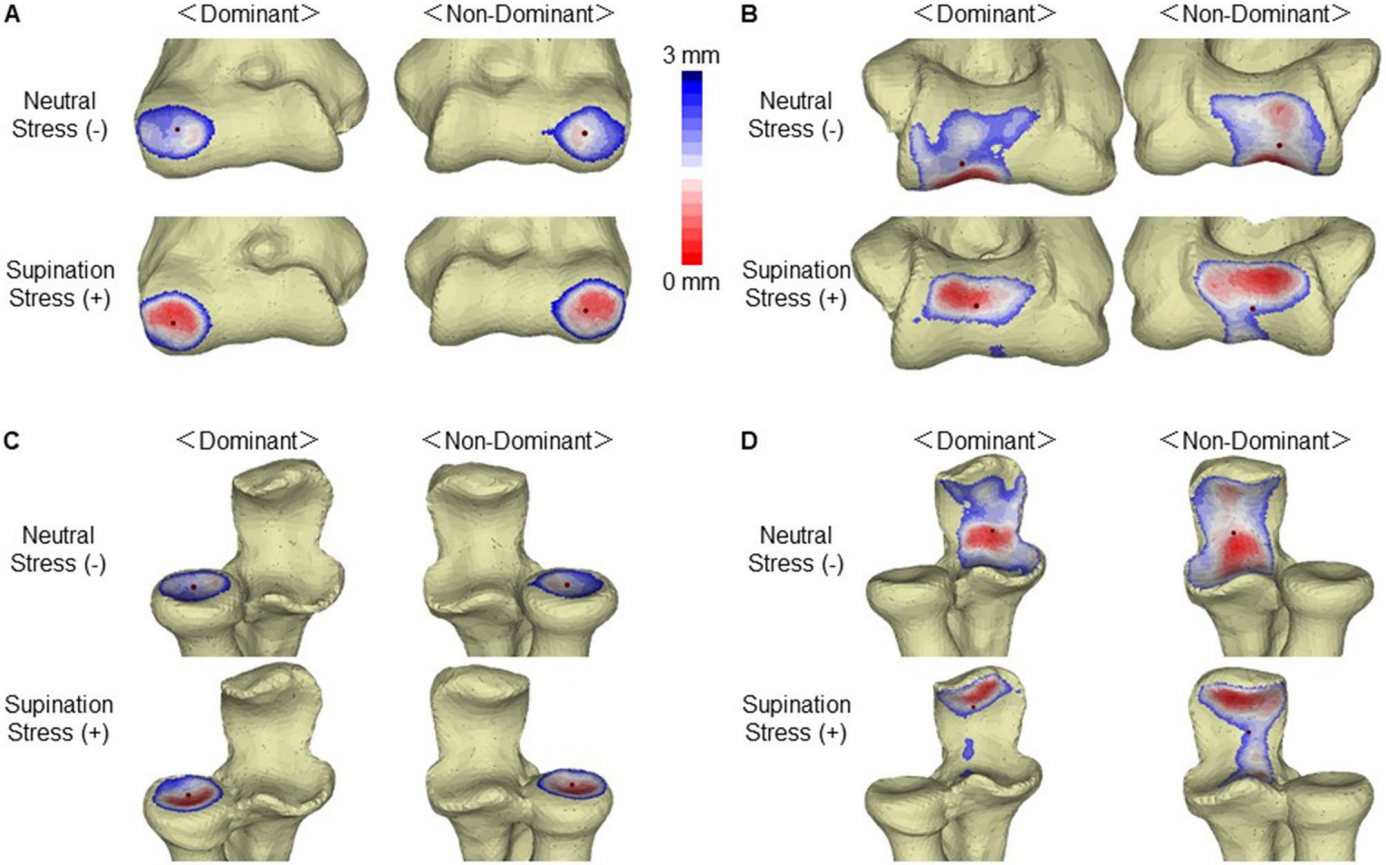
Elbow joint contact area and the centroid of each area. (**A**) The humeral contact area of the radiocapitellar joint. The joint space is wide (blue) and narrow (red). The centroid of each joint contact area is denoted by the black dot. (**B**) The humeral contact area of the ulnohumeral joint. (**C**) The radial contact area of the radiocapitellar joint. (**D**) The ulnar contact area of the ulnohumeral joint.

There was a trend towards increased contact area of the radiocapitellar joint with elbow valgus stress (263.1 ± 40.2 mm^2^) compared to without valgus stress (230.3 ± 32.7 mm^2^) on the non-dominant side (p = 0.0735), but, no difference was found on the dominant side between with valgus stress (240.6 ± 35.0 mm^2^) and without valgus stress (227.9 ± 60.0 mm^2^, p = 0.5901) (Table 2, Fig. 1). There were no significant differences in the mean contact area of the radiocapitellar joint between the dominant and non-dominant sides with and without valgus stress (p = 0.2201 and p = 0.9152, respectively).

**Table 2:** Elbow joint contact area.

Case	Radiocapitellar joint, mm^2^				Ulnohumeral, mm^2^			
	Dominant (D)		Non-dominant (ND)		Dominant		Non-dominant	
	Stress (+)	Stress (−)	Stress (+)	Stress (−)	Stress (+)	Stress (−)	Stress (+)	Stress (−)
1	291.0	326.7	337.3	266.4	529.1	1044.2	663.6	872.8
2	282.9	251.7	262.9	203.3	319.5	838.4	600.5	853.6
3	293.3	290.8	308.7	276.0	652.3	992.8	801.8	821.2
4	227.6	244.5	257.6	215.9	615.8	878.6	796.7	899.4
5	213.9	212.3	281.6	240.5	598.2	765.0	676.6	732.0
6	196.3	138.5	199.7	188.8	387.5	772.0	434.4	731.0
7	207.9	113.8	203.9	172.8	585.9	639.9	613.7	823.1
8	210.4	225.7	279.9	261.9	285.9	739.7	637.5	733.3
9	253.8	238.4	252.1	245.2	528.1	979.2	772.2	923.6
10	228.5	236.2	247.5	232.4	472.9	879.1	718.5	824.9
Mean	240.6	227.9	263.1	230.3	497.5	852.9	671.7	821.5
P value D vs ND	0.2201	0.9152			0.0044	0.5033		
P value Stress (+) vs (−)	0.5901		0.0735		< 0.0001		0.0020

The contact area of the ulnohumeral joint on the dominant side decreased with valgus stress (497.5 ± 121.2 mm^2^, p < 0.0001) to 58.3% of that without valgus stress (852.9 ± 120.9 mm^2^). The contact area of the ulnohumeral joint on the non-dominant side also decreased with valgus stress (671.5 ± 105.3 mm^2^, p = 0.0020) to 81.8% of that without valgus stress (821.5 ± 66.4 mm^2^). Whereas no difference was found in the contact area of the ulnohumeral joint between the dominant and non-dominant sides without valgus stress (p = 0.5033), the contact area was significantly lower on the dominant side than on the non-dominant side with valgus stress (p = 0.0044).

### Translation on the capitellum

**Figure 2:**
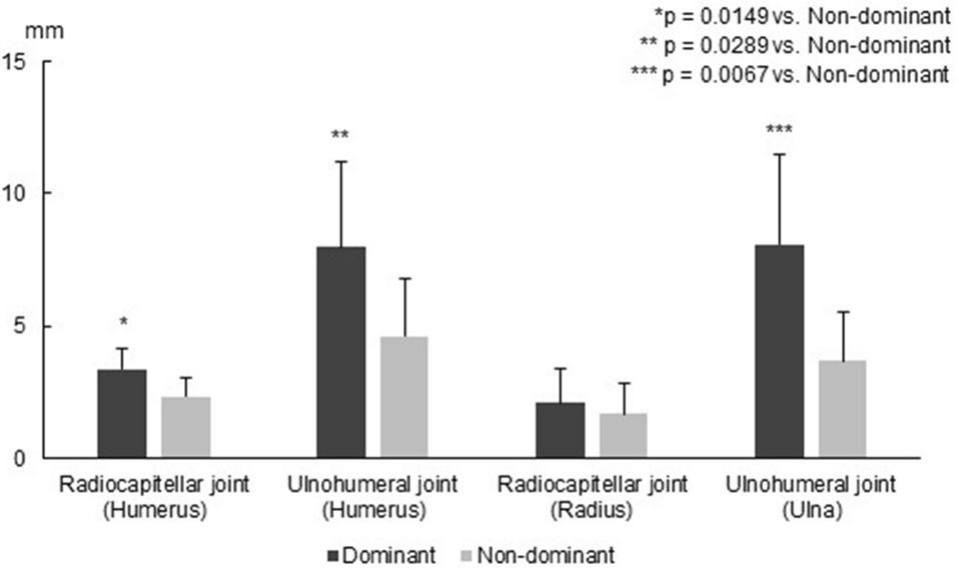
The translation distance of the joint contact area centroid from the position without elbow valgus stress to the position with elbow valgus stress.

**Figure 3:**
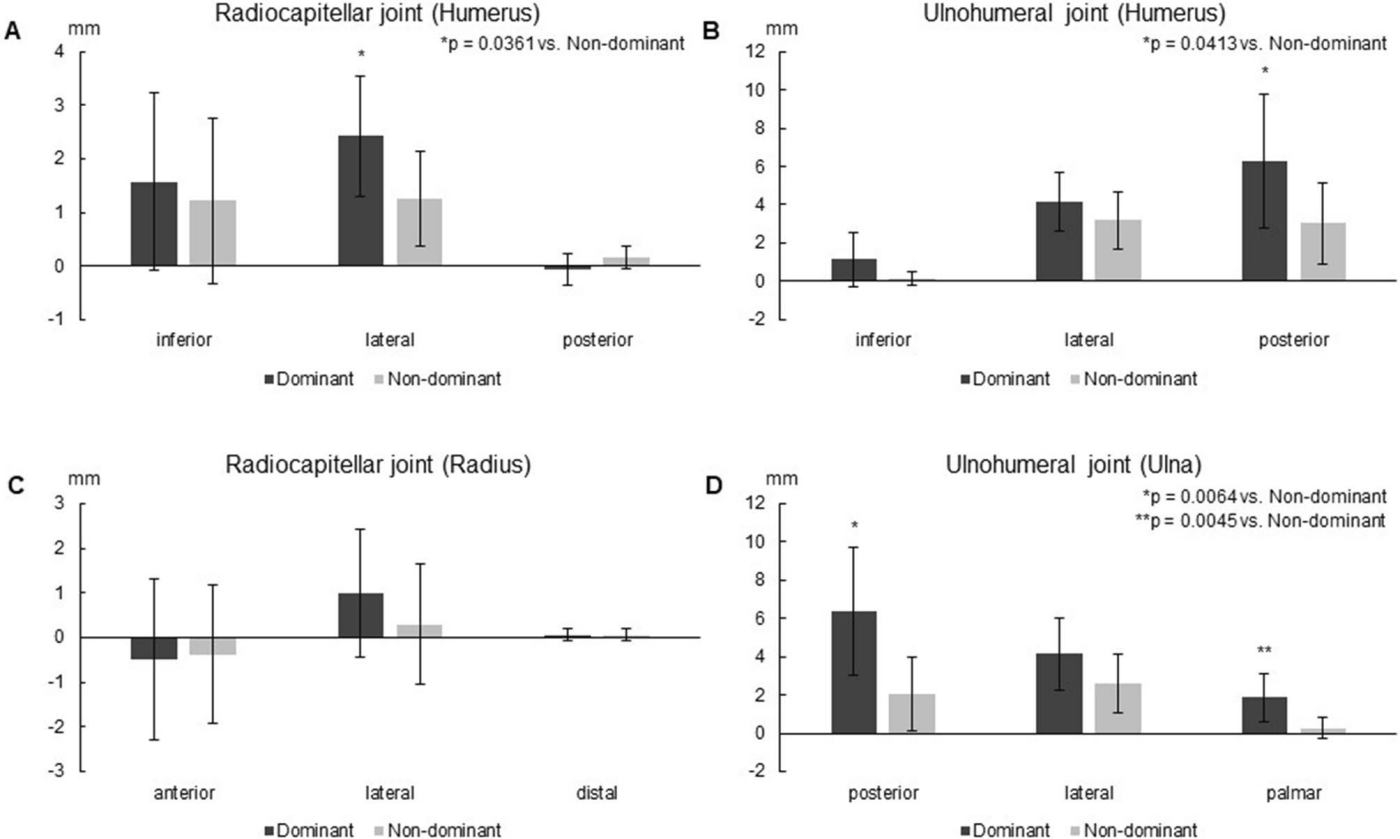
The translation direction of the joint contact area centroid from the position without stress to the position with stress. (**A**) The translation direction of the centroid of the humeral contact area of the radiocapitellar joint from the position without elbow valgus stress to the position with elbow valgus stress. (**B**) The centroid of the humeral contact area of the ulnohumeral joint. (**C**) The centroid of the radial contact area of the radiocapitellar joint. (**D**) The centroid of the ulnar contact area of the ulnohumeral joint.

The total translation distance of the humeral contact area of the radiocapitellar joint centroid was greater on the dominant side (3.37 ± 0.79 mm) than on the non-dominant side (2.35 ± 0.67 mm, p = 0.0149) (Fig. 2). When the translation was decomposed into inferior, lateral, and posterior directions, the lateral translation distance of the humeral contact area of the radiocapitellar joint centroid was larger on the dominant side (2.42 ± 1.11 mm) than on the non-dominant side (1.25 ± 0.89 mm, p = 0.0361) (Fig. 3A).

### Translation on the trochlea

The total translation distance of the humeral contact area of the ulnohumeral joint centroid was greater on the dominant side (7.98 ± 3.22 mm) than on the non-dominant side (4.62 ± 2.19 mm, p = 0.0289) (Fig. 2). When the translation was decomposed into inferior, lateral, and posterior directions, the posterior translation distance of the humeral contact area of the ulnohumeral joint centroid was greater on the dominant side (6.30 ± 3.51 mm) than on the non-dominant side (3.04 ± 2.13 mm, p = 0.0413) (Fig. 3B).

### Translation on the radial head

No significant difference in the total translation distance of the radial contact area of the radiocapitellar centroid was detected between the dominant side (2.11 ± 1.27 mm) and the non-dominant side (1.68 ± 1.13 mm) (Fig. 2). None of the translation distances in the anterior, lateral, and distal directions showed significant differences (Fig. 3C).

### Translation on the trochlear notch

The total translation distance of the ulnar contact area of the ulnohumeral joint centroid was greater on the dominant side (8.07 ± 3.42 mm) than on the non-dominant side (3.69 ± 1.86 mm, p = 0.0067) (Fig. 2). When the translation was decomposed into posterior, lateral, and palmar directions, the posterior translation distance of the ulnar contact area of the ulnohumeral joint centroid was greater on the dominant side (6.38 ± 3.32 mm) than on the non-dominant side (1.85 ± 1.23 mm, p = 0.0064), and the palmar translation distance was greater on the dominant side (2.03 ± 1.92 mm) than on the non-dominant side (0.24 ± 0.55 mm, p = 0.0045) (Fig. 3D).

## Discussion

To the best of our knowledge, this is the first in vivo evaluation of the elbow joint contact area in pitchers with symptomatic valgus instability with elbow valgus stress. The present study demonstrated that the elbow joint contact area differed between the dominant side with symptomatic valgus instability and the non-dominant side without valgus instability. In the pitchers with symptomatic valgus instability with elbow valgus stress, the contact area of the radiocapitellar joint was translated laterally, and that of the ulnohumeral joint was translated posteriorly. The results indicate that, in pitchers with symptomatic valgus instability, stress is most highly concentrated in the lateral part of the radiocapitellar joint and the posterior part of the ulnohumeral joint with elbow valgus stress.

The elbow joint contact area has been recorded in vitro and in vivo using various devices, such as radiographys^9^, cameras^10^, and CT scans^11^. In addition, various experimental methods have been used to study elbow joint contact area, including silicon casting^12^, cartilage staining^13^, pressure-sensitive film technique^14^, and CT scans^15^. However, these studies were based on 2-dimensional images. Furthermore, in in vitro cadaveric studies, the lack of soft tissue tensioning may have affected the normal joint kinematics. In recent years, the in vivo 3D joint contact area has been measured using noninvasive techniques^16^. Using these in vivo 3D methods, Omori et al.^17^ reported that in the neutral position, the contact area of the radiocapitellar joint was 332.5 ± 11.9 mm^2^ and that of the ulnohumeral joint was 1059 ± 40.2 mm^2^. In the present study, the contact areas of the radiocapitellar joint and the ulnohumeral joint with and without elbow valgus stress were investigated using in vivo 3D methods. The current results of the non-dominant elbow without valgus stress are comparable to those of previous reports.

Although several cadaveric studies have analyzed the distribution of the joint contact area through the elbow joint^3,4^, in vitro cadaveric studies lack soft tissue tensioning and internal force applied to the elbow joint which may affect the normal joint kinematics. Thus, 3D CT was used to evaluate changes in contact area through the elbow joint in pitchers with symptomatic valgus instability with elbow valgus stress in the present study, and the biomechanical characteristics of the articular surfaces of the elbow under the in vivo loading conditions were clarified. The present study showed that, in pitchers with symptomatic valgus instability, the radiocapitellar joint contact area was translated laterally, and the ulnohumeral joint was translated posteriorly with elbow valgus stress. Ahmad et al.^4^ reported that medial ulnar collateral ligament deficiency alters the contact area and pressure between the posteromedial trochlea and the olecranon in cadaveric specimens. Change in the contact area was found to occur in pitchers with symptomatic valgus instability with in vivo loading conditions. Furthermore, in pitchers with symptomatic valgus instability with elbow valgus stress, it was shown that the elbow joint contact area of the ulnohumeral joint was translated posteriorly, and that of the radiocapitellar joint was translated laterally. These translations may result in OCD of the capitellum or an olecranon stress fracture.

Posterior elbow joint cartilage injuries are typical in pitchers with symptomatic valgus instability, and they are a severe problem for adolescent pitchers^18^. Osbahr et al.^19^ reported that UCL deficiency rises contact pressures, reduces contact area, and transfers the contact point medially onto the medial crista of the posterior humeral trochlea, may cause chondromalacia at this location. In the early acceleration phase of the throwing motion with the elbow flexed to 90°, the results demonstrate that valgus laxity potentially resulting in abnormal contact through increased contact pressures across the posteromedial elbow between the medial tip of the olecranon and the medial crista of the humeral trochlea. In addition, congruency of the ulnohumeral joint changed, since there was a significant medial shift of the olecranon on the posterior humeral trochlea with the elbow flexed to 90° after sectioning the anterior bundle of the ulnar collateral ligament. The present results of pitchers with symptomatic valgus instability, showing posterior translation in ulnohumeral joint contact with elbow valgus stress, suggest that the posterior elbow cartilage disorder is produced by long-term pitching activities with elbow valgus instability.

The present study had several limitations. First, the contact areas of the radiocapitellar and ulnohumeral joints were estimated from the joint space width distribution at these joints. Although the methods used in the current study allowed comparisons between the symptomatic and non-dominant elbows using the threshold levels reported in the literature to define the contact areas, absolute values of the contact area need to be confirmed by a validation study. Second, the positions of the elbow joint under stress were not true dynamic positions. Nonetheless, the elbows consistently showed a characteristic pattern of elbow kinematic changes and these results appear to successfully represent the elbow kinematics of the symptomatic elbow valgus instability condition.

In conclusion, symptomatic UCL deficiency was associated with a characteristic lateral shift on the anterior part of the capitellum and a posterior shift on the trochlea. These alterations of contact areas could explain the cartilage injury at the posterior trochlea in pitchers with UCL deficiency.

## Methods

### Ethics statement

Our study was carried out in accordance with relevant guidelines of Hokkaido University Hospital and approved by the Research Ethics Review Committee of Hokkaido University Hospital. Our research protocols for human samples used in this study was approved by the Research Ethics Review Committee of Hokkaido University Hospital (approval ID: 020-0087). Informed consents for the use of samples in our research were obtained from all participants. Informed consents for publication of identifying images in an online open-access publication were also obtained.

### Patients and 3D bone model creation

Ten symptomatic baseball pitchers with UCL deficiency and valgus instability participated. There was no obvious traumatic injury, such as dislocation of the elbow joint. Individuals with UCL deficiency with osteochondritis dissecans (OCD) or an olecranon stress fracture were excluded from the current study. All participants were competitive level baseball pitchers in the Japan Amateur Baseball Association. The inclusion criteria for pitchers with symptomatic valgus instability were an inability to throw at full velocity because of medial elbow pain and the presence of elbow laxity on clinical examination and on diagnostic magnetic resonance imaging (MRI) compared with the contralateral elbow. Six-stage MRI-based classification was used to evaluate UCL damage^20^. The pitchers’ weight and height were measured, and the mean number of years on the baseball team was determined. The passive forearm range of motion (ROM) was determined in pronation and supination at 0° of shoulder abduction with elbow flexion of 90° for the dominant and nondominant elbows using goniometer. In addition, the passive elbow ROM was measured in extension and flexion at shoulder abduction of 0°.

**Figure 4:**
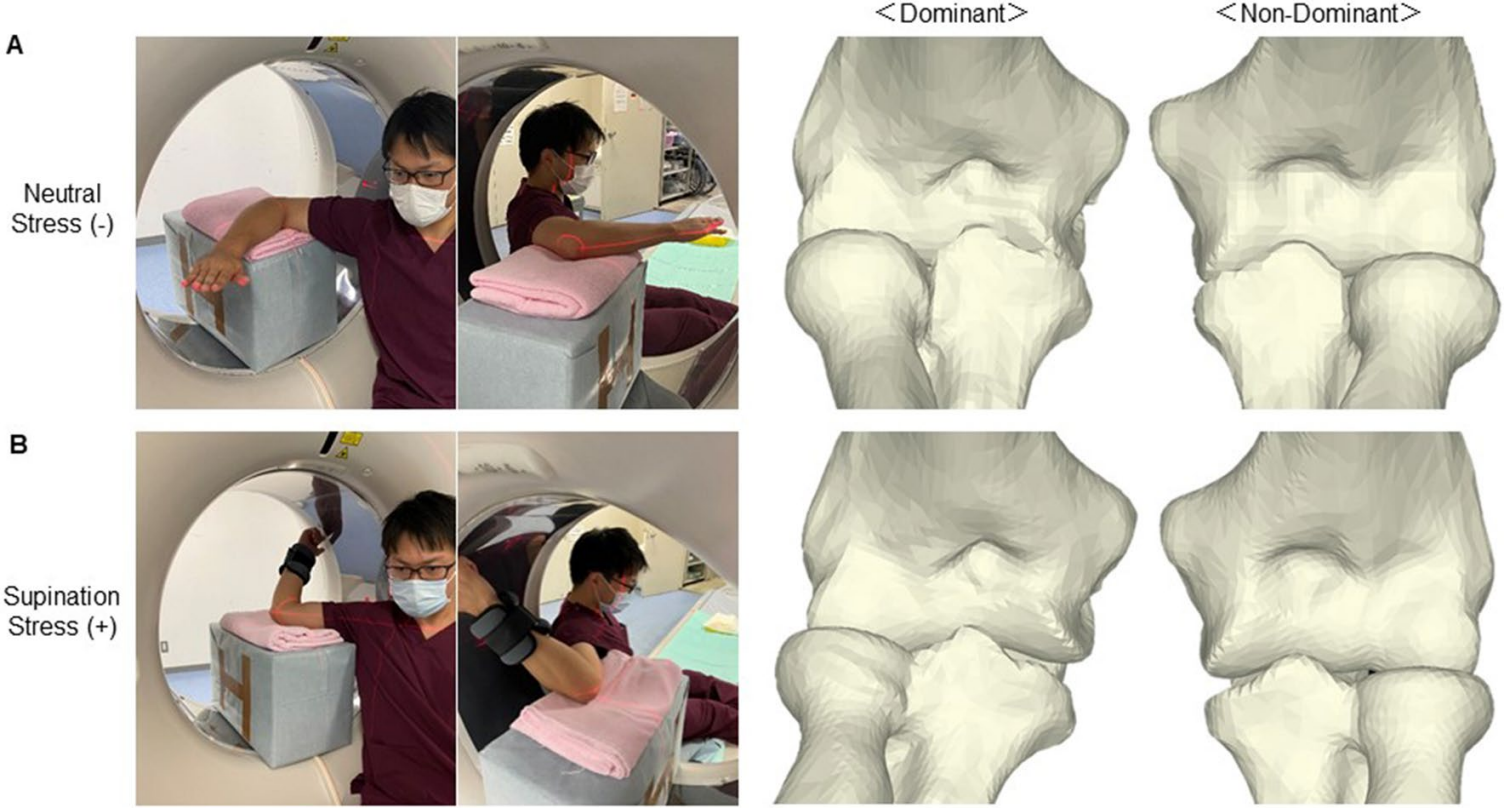
Acquisition of 3D bone models. (**A**) A CT image is obtained with the shoulder in abduction of 90° and the elbow flexed at 90° without valgus stress. 3D bone models of the humerus, radius, and ulna surfaces are created from each arm position. (**B**) With elbow valgus stress.

The 3D CT scanner was an Aquilion One 320-slice, multidetector, wide field-of-view (FOV) scanner (Canon Medical Systems, Tochigi, Japan; slice thickness, 0.5 mm; slice interval, 0.5 mm; matrix 512 × 512; FOV φ500 mm). Three-dimensional CT data were obtained from the dominant and nondominant elbows with and without elbow valgus stress. CT images were obtained with shoulder abduction of 90° and elbow flexion of 90°. For the position without valgus stress, the forearm and shoulder were in neutral rotation (Fig. 4A). For the position with valgus stress, the forearm was in supination of 90° with the shoulder in maximum external rotation and a 2-kg weight was attached to the wrist using wristband (Fig. 4B). CT images of each elbow joint were imported in DICOM format and segmented using a segmentation software package (Mimics 21R, Materialise, Leuven, Belgium). Three-dimensional images of the humerus, radius, and ulna were reconstructed, and the resulting 3D models were then exported as pointcloud and polygon models using the same software package. The 3D humerus, radius, and ulna bone models were then analyzed with custom-written software created using Microsoft Visual C++ with the Microsoft Foundation Class programming environment (Microsoft, Redmond, WA, USA) for further analysis^21–23^.

### Definition of joint contact area and the anatomical coordinate system

**Figure 5:**
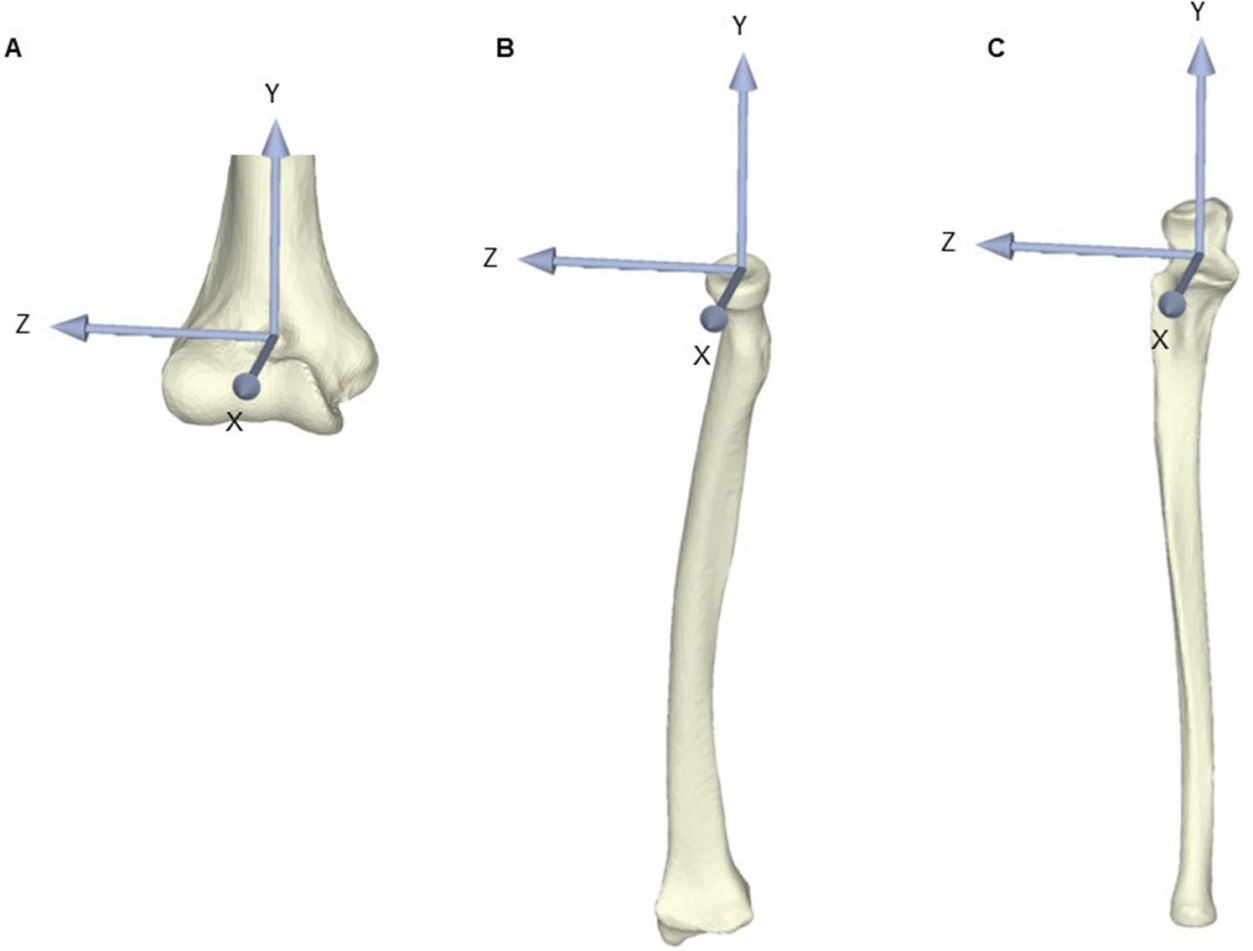
Coordinate system for the direction of translation of the joint contact area centroid. (**A**) The anatomical coordinate systems of the humerus, (**B**) radius, and (**C**) ulna. Rotation around the X-axis, Y-axis, and Z-axis is indicated as varus (+)/valgus (−), internal rotation (+)/external rotation (−), and flexion (+)/extension (−), respectively. Translation along the X-axis, Y-axis, and Z-axis is indicated as the anterior (+)/posterior (−) direction, proximal (+)/distal (−) direction, and lateral (+)/medial (−) direction, respectively.

The surface-to-surface least-distance distributions between the humerus and radius models and between the humerus and ulna models were calculated by a point-to-surface distance calculation algorithm using custom-written software^24^. Articular contact areas were defined as areas where the least distances were under a certain threshold level. The distance thresholds were determined by referencing the previous studies of the distance of the elbow joint space^17^; these thresholds were 2.8 mm in the radiocapitellar joint and 2.4 mm in the ulnohumeral joint. The radiocapitellar and ulnohumeral joint contact areas were calculated from the 3D bone models using custom-written software. The center of the contact area was also calculated, and the translation from neutral to the valgus stress position was also calculated using the custom-written software. To evaluate the translation of the contact area centroid, a validated 3D–3D registration method was used, and a transformation matrix from the original position to the valgus position was obtained^25,26^. The anatomical coordinate system of the elbow was determined using the International Society of Biomechanics standard^27^ (Fig. 5).

### Statistical analysis

An a priori power analysis (G*Power software) indicated that a sample of 10 participants would be appropriate to establish a statistical power of 0.95, at the predetermined a level of 0.05, and with a large effect size of 0.8. The joint contact areas were compared between the dominant and the non-dominant sides using a paired *t-*test. The total translation distance and the decomposed translation distances in anatomical directions defined by the local coordinates of the joint contact area centroid from the position without valgus stress to the position with valgus stress were compared between the dominant and non-dominant sides using a paired *t-*test. p values < 0.05 were considered significant. Data are presented as means ± SD.
